# Development and Validation of Prognostic Models Using Radiomic Features from Pre-Treatment Positron Emission Tomography (PET) Images in Head and Neck Squamous Cell Carcinoma (HNSCC) Patients

**DOI:** 10.3390/cancers16122195

**Published:** 2024-06-11

**Authors:** Mahima Merin Philip, Jessica Watts, Fergus McKiddie, Andy Welch, Mintu Nath

**Affiliations:** 1 Institute of Applied Health Sciences, University of Aberdeen, Aberdeen AB25 2ZD, UK; m.philip.20@abdn.ac.uk; 2 National Health Service Grampian, Aberdeen AB15 6RE, UK; jessica.watts3@nhs.scot (J.W.); fergus.mckiddie@nhs.scot (F.M.); 3 Institute of Education in Healthcare and Medical Sciences, University of Aberdeen, Aberdeen AB25 2ZD, UK; a.welch@abdn.ac.uk

**Keywords:** head and neck squamous cell carcinoma (HNSCC), positron emission tomography (PET), prognosis, radiomics, machine learning

## Abstract

**Simple Summary:**

Time-to-event analysis holds significant relevance for diseases like cancer since accurate disease prognosis is crucial for better patient management and for personalizing treatment. In recent years, survival models using machine learning (ML)-based tools have shown promise in cancer prognosis. We compared four survival models in the ML framework to predict adverse outcomes—all-cause mortality (ACM), locoregional recurrence/residual disease (LR), and distant metastasis (DM)—in head and neck cancer patients. Using radiomic features from pre-treatment positron emission tomography (PET) images, we assessed the performance of these models in an external independent validation cohort. The best-performing model for each outcome was identified based on the highest concordance index and the lowest error in training data. The penalized Cox model for ACM and DM and the random forest model for LR showed promising results. Further training and validation of these models in a larger cohort is required for clinical implementation.

**Abstract:**

High-dimensional radiomics features derived from pre-treatment positron emission tomography (PET) images offer prognostic insights for patients with head and neck squamous cell carcinoma (HNSCC). Using 124 PET radiomics features and clinical variables (age, sex, stage of cancer, site of cancer) from a cohort of 232 patients, we evaluated four survival models—penalized Cox model, random forest, gradient boosted model and support vector machine—to predict all-cause mortality (ACM), locoregional recurrence/residual disease (LR) and distant metastasis (DM) probability during 36, 24 and 24 months of follow-up, respectively. We developed models with five-fold cross-validation, selected the best-performing model for each outcome based on the concordance index (C-statistic) and the integrated Brier score (IBS) and validated them in an independent cohort of 102 patients. The penalized Cox model demonstrated better performance for ACM (C-statistic = 0.70, IBS = 0.12) and DM (C-statistic = 0.70, IBS = 0.08) while the random forest model displayed better performance for LR (C-statistic = 0.76, IBS = 0.07). We conclude that the ML-based prognostic model can aid clinicians in quantifying prognosis and determining effective treatment strategies, thereby improving favorable outcomes in HNSCC patients.

## 1. Introduction

Head and neck squamous cell carcinoma (HNSCC) is a common cancer worldwide, with nearly 900,000 new cases and 500,000 deaths in 2020 alone [1]. Despite advances in medical sciences, the five-year survival rate for HNSCC has not improved over the past three decades. More than 50% of HNSCC patients experience recurrence and distant metastasis within three years of diagnosis. In cases of locally advanced disease, HNSCC has poor prognosis, with a 5-year overall survival rate of less than 50%. To aggravate the issue, treatment options to treat recurrent and metastatic HNSCC are limited compared to other malignancies owing to the disease’s complexity and heterogeneity, contributing to its poor prognosis [2,3,4,5]. Chemotherapy, chemoradiotherapy, targeted therapy and immunotherapy for HNSCC treatments show varying results depending on the age, stage of disease, comorbidities and previous treatment history. The current clinical trials are exploring the promises of molecular targeted therapy and immunotherapy as treatment strategies in HNSCC patients [5]. Given these challenges, early identification of patients with low survival rates is crucial in providing suitable treatment regimens for these patients [6]. However, the accurate prediction of disease prognosis is a challenging task for clinicians due to the complexity of each patient case and increased variability in individual prognostic factors [7,8].

Positron emission tomography (PET) imaging is superior to other imaging modalities in identifying locoregional nodal involvement or distant metastasis [9]. PET radiomic features from primary tumors along with clinical variables may be useful in developing robust prognostic models, predicting and stratifying disease risks, and applying patient-specific treatment strategies [10]. Radiomic features allow the quantification of tumor phenotype and provide insight into various aspects of the disease such as the grade of a tumor, histologic and genetic subtype and the predicted outcome [11]. Combining radiomic features with clinical parameters demonstrated complementary predictive value for adverse outcomes in patients with HNSCC [12]. Additionally, identifying predictors associated with outcomes is critical to provide insights into disease development and progression mechanisms [13].

To develop a robust prognostic model in the presence of censored data with the capability to predict time-to-event outcomes, such as all-cause mortality (ACM), locoregional residual/recurrent disease (LR) or distant metastasis (DM), survival models are considered as the most suitable approach [14]. In survival analysis, a key concern is to deal with censored events where the occurrence of the event of interest is not fully observed for all the subjects by the end of the study. When some subjects do not experience the event of interest or are lost to follow-up during the study period, they are labeled as right-censored events [15]. While the standard Cox proportional hazard (CPH) model represents the conventional choice for survival data with censored events [13], it cannot handle large-scale data efficiently [14]. However, numerous advances have been made to improve survival prediction models, leveraging higher volume and dimension of data with enhanced computational feasibilities. Among these models, machine learning (ML) algorithms have shown promising results [7]. ML models can effectively handle high-dimensional, right-censored and heterogeneous data [14]. The application of ML techniques has led to a notable enhancement in cancer outcome prediction, with improvements ranging from 15% to 20% [8]. ML models also offer significant benefits in identifying patterns and discovering relationships between the predictive features and the outcome probabilities [8]. An array of machine learning methods, including kernel methods, gradient boosting and neural networks, are currently available to model survival outcomes [13].

To our knowledge, limited literature has addressed the suitability of ML models with external validations for time-to-event modelling of adverse outcomes in HNSCC patients using pre-treatment FDG (fluorodeoxyglucose)-PET images [16]. Therefore, the objectives of this study were: (a) to develop and compare the performance of different ML algorithms and validate using an external independent cohort, (b) to identify the best-performing model to estimate the survival probability and (c) to explore features influencing the performance of best models for different outcomes in HNSCC patients.

## 2. Materials and Methods

### 2.1. Patient Data

CheckList for EvaluAtion of Radiomics research (CLEAR) was used for reporting the study [17]. The details of the checklist are available in the supplementary material Table S1. Data for this retrospective study were available from the public database, The Cancer Imaging Archive (TCIA). Ethics approval was not required for this study. Two different datasets available on TCIA were used in this study: one for training the model (with internal validation) [18] (denoted as DS1) and another one for independent external validation of the model (denoted as DS2) [19].

Data for DS1 and DS2 were acquired on hybrid PET-CT scanners, and we considered only the pre-treatment PET images from these scanners. Data for DS1 were collected across four hospitals (HMR, HGJ, CHUS, CHUM) in Canada, and FDG was used as the radiopharmaceutical for PET imaging. For the HGJ cohort, the slice thickness resolution of FDG-PET (GE Healthcare-Discovery ST) was 3.27 mm for all patients and the median in-plane resolution was 3.52 × 5.52 mm^2^. For the CHUS cohort, the slice thickness resolution of FDG-PET (Philips-GeminiGXL) was 4 mm for all patients with a median in-plane resolution of 4 × 4 mm^2^. For the HMR cohort, the slice thickness resolution of FDG-PET (GE healthcare-Discovery STE) was 3.27 mm for all patients with a median in-plane resolution of 3.52 × 3.52 mm^2^, and for the CHUM cohort, the slice thickness resolution of FDG-PET (GE healthcare-Discovery STE) was 4 mm with a median in-plane resolution of 4 × 4 mm^2^. For the 102 patients included in DS2, PET images were obtained on GE Medical System’s Discovery RX, Discovery ST, Discovery HR and Discovery STE hybrid PET/CT by injecting 18F-labeled FDG intravenously. The slice thickness resolution for PET was 3.27 mm for all patients and the median in-plane resolution was 5.47 × 5.47 mm^2^. Further imaging protocol details of DS1 and DS2 can be found in [20,21].

The inclusion criteria for DS1 and DS2 were defined as follows. No cases demonstrated proven metastases at the time of presentation in both datasets. Cases included in the analysis had a follow-up period exceeding 5 months and specifically excluded cases where a pre-treatment PET scan was taken before or after 3 months of diagnosis (biopsy).

In this study, we modelled three outcomes with different follow-up periods: all-cause mortality (ACM) for 36 months, locoregional residual/recurrent disease (LR) for 24 months and distant metastasis (DM) for 24 months. Different follow-up periods for these outcomes were considered accounting for the available information in these two datasets. ACM was defined as cases where death occurred due to any cause from the time of imaging. LR was defined as the cases where cancer had remained or recurred in the same place as the original cancer, or the cancer had grown into lymph nodes or tissues near the original disease. DM was defined as cases where cancer had spread to organs or tissues far from the original cancer. These definitions closely align with the definitions provided in [21]. Patients alive/without LR/without DM at the time of analysis were censored at the date of the last follow-up.

### 2.2. Segmentation

Primary tumors on the PET images on both DS1 and DS2 were manually segmented by a radiologist specializing in head and neck imaging (JW) using LIFEx software (version 7.2.3) available at https://www.lifexsoft.org/ (accessed on 9 June 2022). The scanner setting in DS2 differed from DS1. LIFEx does not give feature values for tumors with <64 voxels as evidenced in the literature [22].

### 2.3. Preprocessing

Image preprocessing before feature extraction in both datasets was carried out on the LIFEx software (version 7.2.3). DS1 included data from multiple centers, but feature-level harmonization was not performed due to an insufficient number of samples in one of the cohorts. In PET imaging, if the neighboring voxels are not isotropic, it is necessary to resample them to an isotropic voxel size to make the feature extraction rotationally invariant [23]. The images were spatially resampled to a voxel size of 4 × 4 × 4 using a polynomial of degree 5 (quintic Lagrangian). Intensity discretization is an important preprocessing step to group close gray levels together before feature extraction to reduce the impact of noise [23]. In the context of PET imaging, absolute resampling (discretization) with 64 grey levels (bin size = 0.3125) between 0 and 20 standardized uptake value (SUV) units were used since the majority of SUV values in the training set (DS1) were between 0 and 20 SUV units. Radiomic features were extracted from primary tumors segmented on original images.

### 2.4. Feature Extraction

Hand-crafted features were extracted from the segmented image using LIFEx software. The software calculates a broad range of features in compliance with the Image Biomarker Standardization Initiative (IBSI). Radiomic features were extracted in a three-dimensional (3D) space. In total, 151 features across six feature classes (shape, first order, gray level co-occurrence matrix (GLCM), gray level run length matrix (GLRLM), neighborhood gray-tone difference matrix (NGTDM), and gray level size zone matrix (GLSZM)) were extracted from the PET images. After removing non-informative features (features with null values, features with similar values for all cases, feature values distributed in x, y, z axes, and feature values spread across different layers), 124 radiomic features were included in the final model development. The details of the radiomic features included in the analysis and the summary statistics of these features in DSI are provided in the Supplementary material (Tables S2 and S3). In addition to radiomics features, the model also included age, sex, stage of cancer and site of the primary tumor. The non-radiomics features were defined in the protocol details of DS1 and DS2 [20,21]. Due to limitations with sample sizes and to ensure that DS1 and DS2 have similar distributions of cancer sites and stages, some site and stage categories were combined.

### 2.5. Data Preparation

After the extraction of features, we developed a pipeline within the Python programming environment to facilitate the stages from data preparation to model development. The pipeline encompassed feature scaling, feature selection, model training, model evaluation and external validation. The pipeline predominantly employed Python (version 3.9.7) packages such as scikit-learn (version 0.24.2) [24] for data preparation and scikit-survival (version 0.16.0) [25] for developing, evaluating and validating survival models.

Since all segmentation tasks were conducted by a single operator, we did not conduct any segmentation reliability analysis. None of the features included in the analysis had any missing values. For the numeric radiomic features of DS1, feature scaling was performed using the min–max scaling technique. Categorical variables were transformed through one hot encoding. To reduce overfitting and to ensure robustness and generalizability of the developed models, we employed a robust feature selection strategy including hyperparameter optimization and cross validation, the details of which are provided in the following sections.

**Figure 1:**
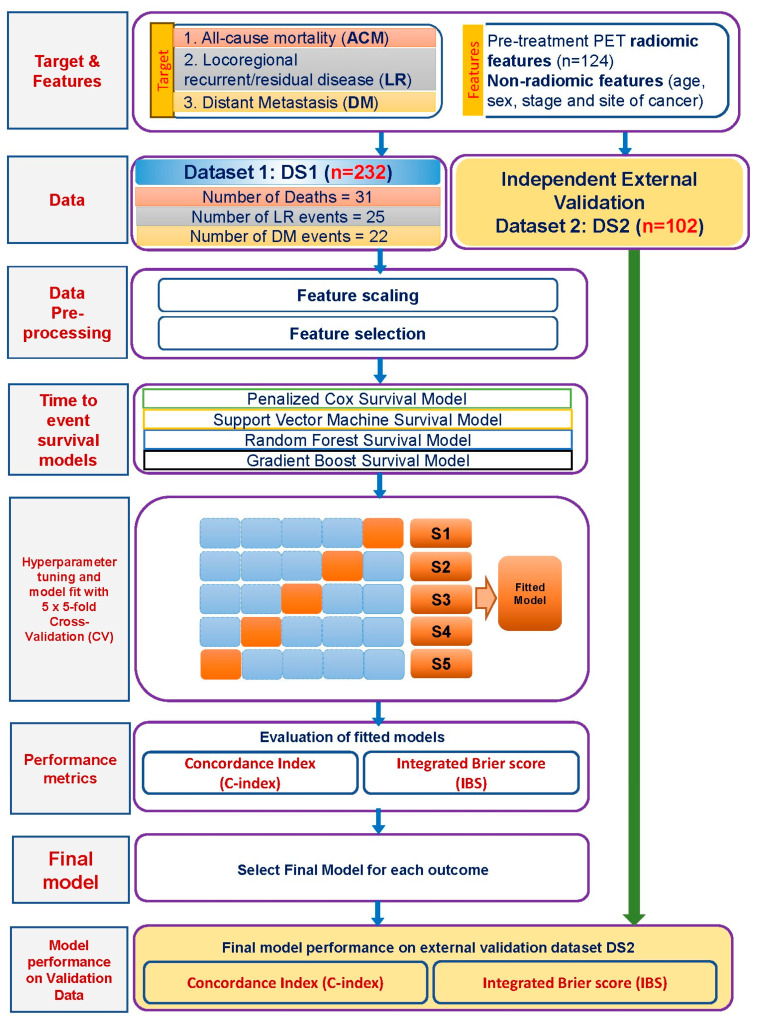
Flow diagram depicting the data structure and machine learning pipeline for fitting the time-to-event survival models.

Feature selection was implemented by assessing the correlation coefficients between features—with highly correlated features excluded using a threshold of 0.95—which gave the best performance in a cardiac study [26]. Following the feature selection, we incorporated 65 radiomic features in the final model development stage. The correlation heatmaps for different classes of features are provided in the Supplementary Material (Figures S1–S6). An overview of the entire modelling pipeline is presented in Figure 1.

### 2.6. Model Training and Validation

The scikit-survival [25] Python module for survival analysis was used to model time-to-event outcomes (ACM, LR and DM). The four algorithms evaluated for fitting survival models on the data were: penalized Cox model, random forest, gradient boosted model, and support vector machine. Detailed descriptions of these models are available in the literature [27,28,29,30]. Observed survival estimates for all outcomes were presented using the Kaplan–Meier plots. The survival models considered the right-censored survival data with each time-to-event data containing a survival time (time between imaging and the event) and a status indicator (event observed or censored) [13].

Hyperparameter tuning was conducted using the randomized search cross-validation. The final model in the training set (DS1) was selected after five-fold cross-validation. To assess the contribution of each feature to the performance of the fitted model, permutation feature importance was estimated in the training set (DS1) by repeatedly shuffling each feature 1000 times [31]. The entire cohort of DS1 was used for model training. External validation of the model was performed using DS2 to assess the generalizability of the model.

### 2.7. Model Performance Evaluation

The model performances were evaluated using Harrell’s concordance index (C-index) to measure the discriminatory power and integrated Brier scores (IBS) to assess the accuracy of the predicted survival function and model calibration [32]. A C-index value of 1 signifies perfect discrimination and 0.5 indicates random prediction. IBS range from 0 to 1 represents perfect and worst discrimination and calibration, respectively. A model with a Brier score below 0.25 indicates a potentially useful model [32]. Permutation feature importance identifies the feature with the highest feature importance, indicating that the feature has the greatest impact on the model’s performance [31]. The model presenting the best performance metrics for each outcome based on the internal validation (i.e., following five-fold cross-validation) was selected as the final model. IBS does not apply to the support vector machine survival model since it does not provide an estimate of the survival function [33].

## 3. Results

### 3.1. Patient Characteristics

The characteristics of patients included in the study are summarized in Table 1. DS1 was a Canadian-based cohort with 232 patients (182 men and 50 women; mean age 63.03 ± 10.38 years), and DS2 was a US-based cohort with 102 patients (90 men and 12 women; mean age, 58.18 ± 8.96 years). In both DS1 and DS2, the major site of the tumor was the oropharynx: in DS1 71.55% and in DS2, 73.55%. The preferred treatment modality in both datasets was chemoradiotherapy with or without surgery. In DS1, 31 patients did not survive beyond 36 months, 25 patients developed locoregional recurrence or residual disease within 24 months, and 22 patients had distant metastasis within 24 months of follow-up.

### 3.2. Machine Learning-Based Prognostic Models

We considered a total of 65 prognostic radiomics and four clinical features for each time-to-event outcome (ACM, LR, DM) to train four models. Table 2 presents the performance metrics of all models in internal and external validation data for all outcome variables.

#### 3.2.1. All-Cause Mortality

**Figure 2:**
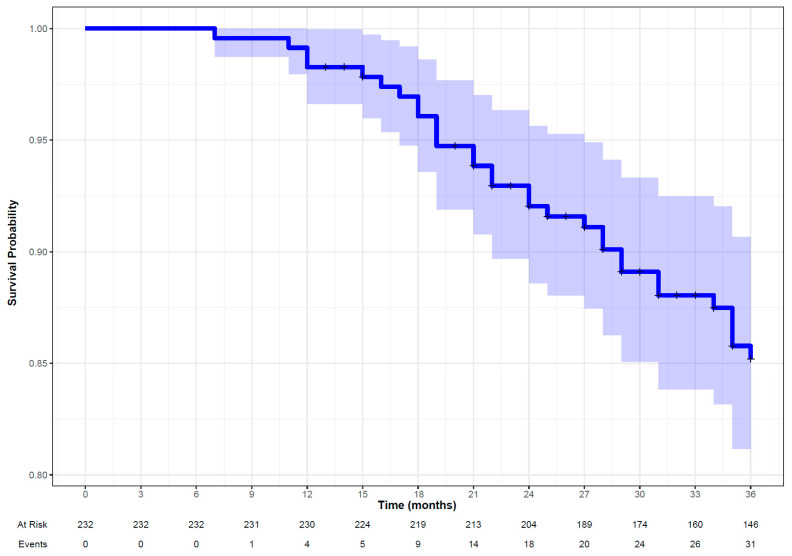
Kaplan–Meier curve along with patient at risk and number of observed events (all-cause mortality) in patients with head and neck squamous cell carcinoma followed up for 36 months.

**Figure 3:**
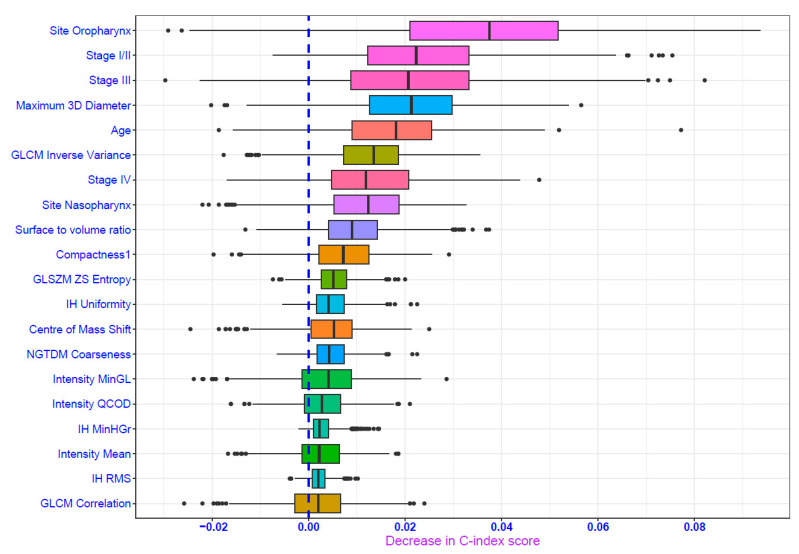
Permutation feature importance plot depicting important predictors for penalized Cox model for all-cause mortality (ACM) outcome. IH: Intensity Histogram; GLCM: Gray Level Cooccurrence Matrix; GLSZM: Gray Level Size Zone Matrix; NGTDM: Neighborhood Gray Tone Difference Matrix; ZSEntropy: Zone Size Entropy; MinGL: Minimum Gray Level; QCOD: Quartile Coefficient of Dispersion; MinHGr: Minimum Histogram Gradient; RMS: Root Mean Square.

The penalized Cox model was selected as the best-performing model based on performance metrics to predict ACM. It demonstrated a reasonable performance in the validation set (DS2) with a C-index of 0.70 and an IBS of 0.12. Random forest and support vector machine also showed comparable performances (Table 2). The Kaplan–Meier curve in Figure 2 suggests that the survival probability was more than 0.85 at 36 months and displays a higher proportion of censoring than the number of events of interest during the follow-up period. The oropharynx as the site of primary tumor is the significant feature that contributes to the performance of the penalized Cox model alongside shape-based (maximum 3D diameter), GLCM_inverse variance and other clinical features (Figure 3). The hyperparameters for the penalized Cox model are provided in the Supplementary material (Table S4).

#### 3.2.2. Locoregional Recurrent/Residual Disease

**Figure 4:**
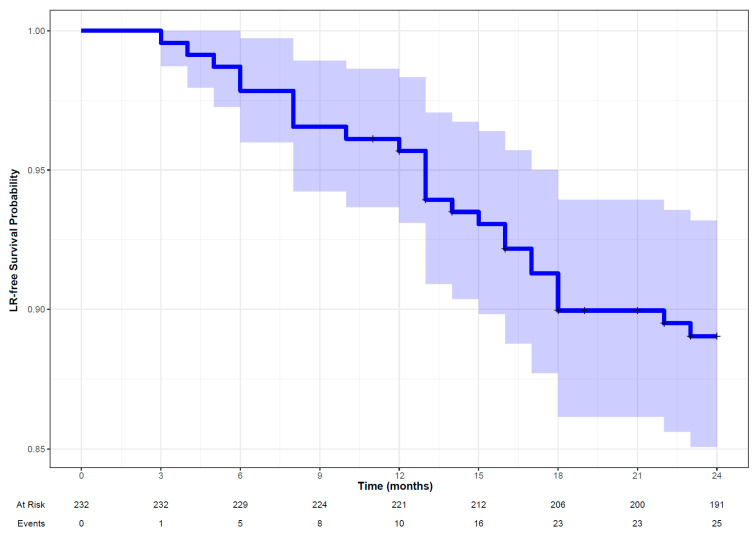
Kaplan–Meier curve along with patient at risk and number of observed events (LR, locoregional recurrent/residual disease) in patients with head and neck squamous cell carcinoma followed up for 24 months.

**Figure 5:**
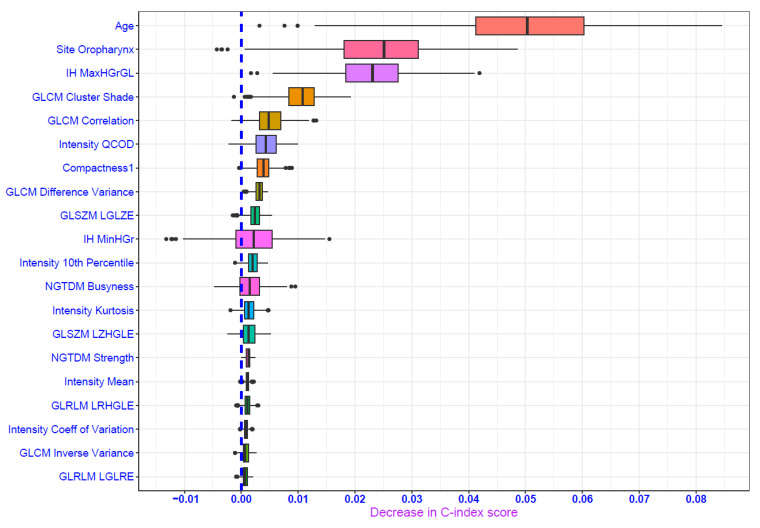
Permutation feature importance plot depicting important predictors for random forest model for locoregional recurrent/residual disease outcome. IH: Intensity Histogram; GLCM: Gray Level Cooccurrence Matrix; GLSZM: Gray Level Size Zone Matrix; NGTDM: Neighborhood Gray Tone Difference Matrix; GLRLM: Gray Level Run Length Matrix; MaxHGrGL: Maximum Histogram Gradient Gray Level; QCOD: Quartile Coefficient of Dispersion; LGLZE: Low Gray Level Zone Emphasis; MinHGr: Minimum Histogram Gradient; LZHGLE: Large Zone High GrayLevel Emphasis; LRHGLE: Long Run High Gray Level Emphasis; LGLRE: Low Gray Level Run Emphasis.

Following five-fold cross-validation and evaluation of performance metrics, the random forest survival model was identified as the best model to predict LR. It also demonstrated good performance in the validation set (DS2) with a C-index of 0.76 and an IBS of 0.07 (Table 2). The Kaplan–Meier curve suggests that the LR-free survival probability was more than 0.875 at 24 months (Figure 4) with an indication of a higher proportion of censoring during the study period as captured by the wider confidence interval around the survival estimates. Age, oropharynx as tumor site, GLCM-based features (clustershade and correlation) and first-order feature (IH_MaxHGrGL: Intensity Histogram Maximum Histogram Gradient Gray Level)) were the significant features contributing to the performance of the random forest model (Figure 5). The hyperparameters for the random forest are provided in the Supplementary material (Table S4).

#### 3.2.3. Distant Metastasis

**Figure 6:**
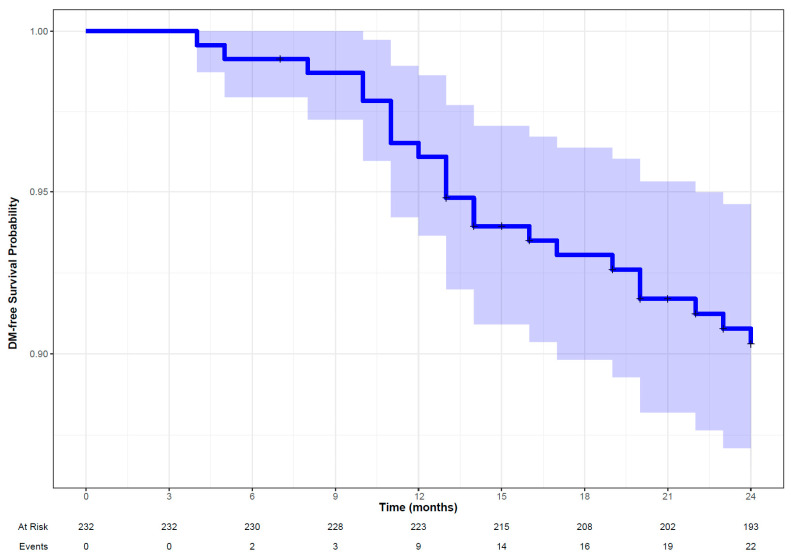
Kaplan–Meier curve along with patient at risk and number of observed events (DM, distant metastasis) in patients with head and neck squamous cell carcinoma followed up for 24 months.

**Figure 7:**
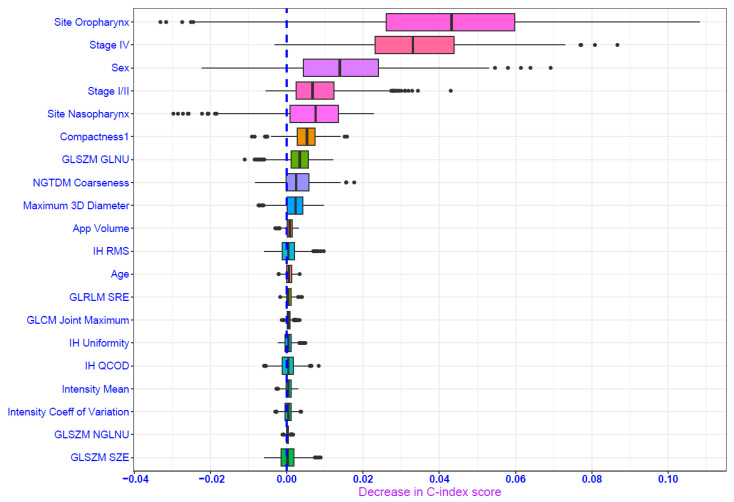
Permutation feature importance plot depicting important predictors for penalized Cox model for distant metastasis outcome. IH: Intensity Histogram; GLCM: Gray Level Cooccurrence Matrix; GLSZM: Gray Level Size Zone Matrix; NGTDM: Neighborhood Gray Tone Difference Matrix; GLRLM: Gray Level Run Length Matrix; GLNU: Gray Level Non-Uniformity; AppVolume: Approximate Volume; RMS: Root Mean Square; SRE: Short Run Emphasis; QCOD: Quartile Coefficient of Dispersion; NGLNU: Normalized Gray Level Non Uniformity; SZE: Small Zone Emphasis.

The penalized Cox model was selected as the best-performing model for distant metastasis (DM). It had a reasonably better performance in the validation set (DS2) with a C-index of 0.70 and an IBS of 0.08 (Table 2). The Kaplan–Meier curve suggests that DM-free survival probability was more than 0.90 at 24 months (Figure 6) displaying a wider confidence interval of the survival estimates later in the period due to an increased proportion of censoring events. Clinical features (site of the primary tumor, sex, stage of cancer) were the significant features contributing to the performance of the penalized Cox model in addition to shape-based compactness, GLSZM_GLNU (GLSZM Gray-Level Non-Uniformity) and NGTDM_Coarseness (Figure 7). The hyperparameters for the penalized Cox model are provided in the Supplementary material (Table S4).

## 4. Discussion

Employing a robust ML-based modelling approach combined with external model validation, our study highlights the potential utility of PET radiomics features in predicting adverse outcomes in patients with HNSCC. Our findings suggest that, for predicting ACM and DM in HNSCC using PET radiomics, the penalized Cox model performs better, while the random forest model exhibits favorable performance for LR. The time-to-event models hold particular significance within the context of HNSCC as the 5-year survival rate in this malignancy remains below 50% and prognosis is influenced by factors like disease stage, locoregional recurrence and distant metastasis [34]. From the PET radiomic features, information about tumor heterogeneity could be assessed, that expands on the information available from laboratory tests, clinical reports and genomic or proteomic assays [35]. Radiomic features, when used for machine learning modelling, macroscopically decode the phenotype of pathophysiological structures, providing valuable insights about disease diagnosis and prognosis, thereby suggesting its correlation with tumor biology [35,36]. For example, for the radiomic feature NGTDM_Coarseness that quantifies tumor granularity, a coarse texture indicates a high degree of local uniformity in intensity, whereas for NGTDM_Busyness, a busy texture implies rapid changes in intensity from one pixel to the neighboring pixel [37,38]. In head and neck cancer NGTDM coarseness and busyness can differentiate primary tumor and lymph nodes from healthy tissues [38]. The application of FDG-PET radiomics-based ML models may play a pivotal role in HNSCC management, assisting clinicians in predicting the recurrence risk and other adverse outcomes, and hence facilitating patient stratification for tailored treatments [39].

When compared with other models, we observed that the penalized Cox model performed reasonably well in predicting ACM and DM in independent datasets. These findings are consistent with previous HNSCC prognostic studies, where the Cox proportional hazard and the random forest models showed comparable performances in external validation for ACM [16]. There are, however, reports suggesting that the random forest survival model for DM prediction achieved better performance in external validation cohorts [16]. The penalized Cox model’s satisfactory performance for these outcomes could be due to its ability to enhance the predictive ability by effectively increasing the number of events per feature through the regularization technique of shrinking the regression parameters towards zero. The penalized Cox model proves particularly effective in high-dimensional survival data settings [40]. A study on oropharyngeal carcinoma reported that the penalized Cox model with radiomic features performed better in the external validation cohort for survival prediction [41].

We observed that the random forest model performed better in predicting LR. However, one study indicated that the Cox proportional hazard model had better performance in the external validation cohort for LR outcome prediction [16]. The superior performance of the random forest survival model could be attributed to its property of handling outliers and right-censored data, its ability to accommodate interactions between variables and non-linearity, and its non-dependence on the proportionality assumption [42,43].

All selected survival models reported in this study demonstrated acceptable estimates for concordance index (C-index) and integrated Brier score (IBS) both on training and validation datasets. Assessing the predictive performance of a survival model is important to determine the quality of the developed model [44]. The C-index is a widely used performance evaluation metric in the majority of the reported studies focusing on HNSCC PET [16]. The C-index is a valuable tool suggesting the performance of the model to discriminate between patients based on their risk. On the other hand, the Brier score is used to evaluate the overall performance of survival models, measuring both calibration and discrimination at a given time point. The IBS provides the average Brier score over a specified time period [45].

Our study, based on the permutation feature importance, suggested that clinical features in addition to shape-based and second-order features were the most important for the predictive model setting. Interestingly, we did not observe strong evidence of the importance of SUV-based features as reported [46]. This could be due to the methodology used to identify important features in ML models. Permutation feature importance, a model agnostic technique, reflects how important a feature is to the model rather than the predictive value of a feature to the outcome [31]. Notwithstanding, we noted similar features were reported to be predictive in other studies. Different HNSCC PET radiomic studies reported a range of features important for predicting different outcomes. Significant predictors for predicting ACM were reported as age, tumor site, T-stage, as well as shape-based and GLCM-based features. Recurrence prediction, on the other hand, was associated with age, tumor volume, and second-order radiomic features. Typical features for predicting DM are clinical variables [16]. It is worth noting that these studies primarily employed the Cox proportional hazards model with a limited number of features to identify key predictors of outcomes. Currently, there exist challenges to assess the statistical significance of predictive variables in non-linear ML models and to compare them with the inferential framework of the standard multivariable Cox survival model, which provides estimates of effect size and their statistical significance [47].

### Limitations and Recommendations

While this study offers a comprehensive comparison of machine learning methods for HNSCC prognosis incorporating time-to-event data, it is not without limitations. Firstly, the retrospective nature of the study resulted in the exclusion of several clinical variables such as HPV (human papilloma virus) status and smoking status that could potentially serve as predictors of the outcome of interest, highlighting the need for a prospective study. Secondly, despite external validation of the calibrated model, the machine learning models were trained on a relatively small dataset without conducting an appropriate sample size calculation, as recommended for modelling studies [48]. The small sample size also hindered the development of site specific and stage specific HNSCC survival models which would otherwise provide added insights to the clinicians. Our study was constrained by the availability of publicly available resources. This study solely relied on radiomics features derived from pre-treatment PET images; however, adopting a multi-omics approach related to HNSCC could improve patient management demonstrating promising results [49,50]. Furthermore, implementing the Combat harmonization tool to address multicenter data variability and accounting for imaging protocol effects may further enhance the performance of the prognostic models [51,52]. Our study involved only a single radiologist for manual tumor segmentation. However, recognizing the possibility of inter-operator variability, it is recommended to include multiple radiologists for tumor segmentation to ensure reliability and reproducibility of segmentation results, by following a clear protocol [53]. Finally, we explored only a selected set of machine learning models to identify the best-performing model, while several other machine learning or deep learning algorithms for time-to-event analysis have been recently reported [54,55].

## 5. Conclusions

This study evaluated the performance metrics of four time-to-event machine learning algorithms for the prediction of ACM, LR and DM in HNSCC using multi-dimensional PET radiomics features, identified the best model and validated the model on an external dataset. For ACM and DM, the penalized Cox model, and for LR, the random forest model exhibited better performance compared to the other models. Further training and validations of the models in a larger cohort, adopting a multi-omics approach and inclusion of other clinical features, are required to facilitate personalized treatment planning in clinical practice.

## Figures and Tables

**Table 1:** Characteristics of patients in the training (Dataset 1) and external validation cohort (Dataset 2).

Variable	Summary	Estimate (Dataset 1)	Estimate (Dataset 2)
Number of patients	Total	232	102
Sex	Male	182	90
	Female	50	12
Age (years)	Overall: Mean (SD)	63.03 (10.38)	58.18 (8.96)
Primary site [*n* (%)]	Oropharynx	166 (71.55)	75 (73.53)
	Nasopharynx	22 (9.48)	4 (3.92)
	Hypopharynx	8 (3.44)	6 (5.88)
	Others (Larynx, Glottis, Oral cavity, Sinus)	36 (15.52)	17 (16.67)
Stage of cancer [*n* (%)]	Stage I/II (II, IIB)	24 (10.34)	4 (3.92)
	Stage III	55 (23.71)	16 (15.69)
	Stage IV (IV, IVA, IVB)	153 (65.95)	82 (80.39)
Treatment strategy [*n* (%)]	Chemoradiation with/without surgery	196 (84.48)	57 (55.88)
	Chemoradiation with/without surgery and targeted therapy	-	7 (6.86)
	Radiation with/without surgery	36 (15.52)	25 (24.51)
	Radiation with/without surgery and targeted therapy	-	13 (12.75)
Number [*n* (%)]	All-cause deaths	31 (13.36)	21 (20.59)
	Locoregional recurrent/residual disease	25 (10.78)	14 (13.73)
	Distant metastasis	22 (9.48)	12 (11.76)
Follow-up time in months [Median (Interquartile range)]	All-cause mortality (ACM)	41 (25.96)	63 (48.99)
	Locoregional recurrent/residual disease (LR)	38 (26.38)	53 (60.31)
	Distant metastasis (DM)	39 (27.43)	53 (60.31)

**Table 2:** Performance comparison of various prognostic models in the training cohort (DS1) and external validation cohort (DS2).

Outcome of Interest	Model	Concordance Index (CI)		Integrated Brier Score (IBS)	
		Internal Validation (Dataset 1)	External Validation (Dataset 2)	Internal Validation (Dataset 1)	External Validation (Dataset 2)
All-cause mortality (ACM)	Penalized Cox	0.78	0.70	0.05	0.12
	Random forest	0.72	0.70	0.05	0.12
	Gradient boosted model	0.78	0.66	0.06	0.12
	Support vector machine	0.81	0.70	NA	NA
Locoregional recurrent/residual disease (LR)	Penalized Cox	0.84	0.66	0.05	0.07
	Random forest	0.86	0.76	0.05	0.07
	Gradient boosted model	0.79	0.61	0.06	0.07
	Support vector machine	0.82	0.65	NA	NA
Distant metastasis (DM)	Penalized Cox	0.79	0.70	0.04	0.08
	Random forest	0.76	0.70	0.04	0.08
	Gradient boosted model	0.79	0.71	0.04	0.09
	Support vector machine	0.77	0.71	NA	NA

NA = Not available.

## Data Availability

Primary PET image data used in this study are available to download in The Cancer Imaging Archive (TCIA) (https://www.cancerimagingarchive.net/) (accessed on 1 February 2023). The secondary feature data are available on request.

## Supplementary Materials

The following supporting information can be downloaded at: https://www.mdpi.com/article/10.3390/cancers16122195/s1. Table S1: CLEAR checklist without explanations. Table S2: List of LIFEx (v7.2.3) features used for analysis. Table S3: Summary statistics of radiomic features in dataset 1 (training set). Table S4: Hyperparameters for the best model used for external validation for all outcomes. Figure S1: Correlation heatmap for age and shape-based features. Figure S2: Correlation heatmap for first-order features. Figure S3: Correlation heatmap for gray level co-occurrence matrix (GLCM) features. Figure S4: Correlation heatmap for gray level run length matrix (GLRLM) features. Figure S5: Correlation heatmap for neighborhood gray-tone difference matrix (NGTDM) features. Figure S6: Correlation heatmap for gray level size zone matrix (GLSZM) features.
