# Supplementary material for: Development and Validation of Prognostic Models Using Radiomic Features from Pre-Treatment Positron Emission Tomography (PET) Images in Head and Neck Squamous Cell Carcinoma (HNSCC) Patients
Source: Cancers (Basel). 2024 Jun 11;16(12):2195. doi: 10.3390/cancers16122195 (PMC11202084; doi:10.3390/cancers16122195)
Supplement: Supplementary file 1 [file cancers-16-02195-s001.zip › cancers-3039708-supplementary.pdf]

Supplementary Material

Development and validation of prognostic models using radiomic features from pre-treatment Positron Emission Tomography (PET) images in Head and Neck Squamous Cell Carcinoma (HNSCC) patients

Mahima Merin Philip[1], Jessica Watts[2], Fergus McKiddie[2], Andy Welch[3], Mintu Nath[1*]

[1]Institute of Applied Health Sciences, University of Aberdeen, AB25 2ZD, UK

[2]National Health Service Grampian, Aberdeen AB15 6RE, UK

[3]Institute of Education in Healthcare and Medical Sciences, University of Aberdeen, AB25 2ZD, UK

[*]Corresponding author (mintu.nath@abdn.ac.uk)

Contents

Abbreviations....................3

Table S1: CLEAR checklist without explanations....................4

Table S2: List of LIFEx (v7.2.3) features used for analysis....................8

Table S3: Summary statistics of radiomic features in dataset 1 (training set)....................11

Table S4: Hyperparameters for the best model used for external validation for all outcomes....................16

Figure S1: Correlation heatmap for age and shape-based features....................17

Figure S2: Correlation heatmap for first-order features....................18

Figure S3: Correlation heatmap for gray level co-occurrence matrix (GLCM) features....................19

Figure S4: Correlation heatmap for gray level run length matrix (GLRLM) features....................20

Figure S5: Correlation heatmap for neighborhood gray-tone difference matrix (NGTDM) features....................21

Figure S6: Correlation heatmap for gray level size zone matrix (GLSZM) features....................22

Abbreviations

**HNSCC**	Head and Neck Squamous Cell Carcinoma
**PET**	Positron Emission Tomography
**FDG**	Fluorodeoxyglucose
**ML**	Machine Learning
**CLEAR**	CheckList for EvaluAtion of Radiomics research
**TCIA**	The Cancer Imaging Archive
**IBSI**	Image biomarker standardisation initiative
**DS1**	Dataset 1
**DS2**	Dataset 2
**ACM**	All-cause mortality
**LR**	Locoregional residual/recurrent disease
**DM**	Distant Metastasis
**3D**	Three dimensional
**SUV**	Standardised uptake value
**GLCM**	Gray level cooccurrence matrix
**GLRLM**	Gray level run length matrix
**NGTDM**	Neighborhood gray-tone difference matrix
**GLSZM**	Gray level size zone matrix
**CPH**	Cox Proportional Hazard
**CI**	Concordance Index
**IBS**	Integrated Brier Score
**SD**	Standard deviation
**HPV**	Human papillomavirus

Table S1: CLEAR checklist without explanations

Section	No	Item	Yes	No	n/a or n/e
Title					
	1	Relevant title, specifying the radiomic methodology	√		
Abstract					
	2	Structured summary with relevant information	√		
Keywords					
	3	Relevant keywords for radiomics	√		
Introduction					
	4	Scientific or clinical background	√		
	5	Rationale for using a radiomic approach	√		
	6	Study objective(s)	√		
Method					
Study Design	7	Adherence to guidelines or checklists (e.g., CLEAR checklist)	√		
	8	Ethical details (e.g., approval, consent, data protection)	√		
	9	Sample size calculation	√		
	10	Study nature (e.g., retrospective, prospective)	√		
	11	Eligibility criteria	√		
	12	Flowchart for technical pipeline	√		
Data	13	Data source (e.g., private, public)	√		
	14	Data overlap			n/a
	15	Data split methodology			n/a
	16	Imaging protocol (i.e., image acquisition and processing)	√		

Section	No	Item	Yes	No	n/a or n/e
	17	Definition of non-radiomic predictor variables	√		
	18	Definition of the reference standard (i.e., outcome variable)	√		
Segmentation	19	Segmentation strategy	√		
	20	Details of operators performing segmentation	√		
Pre-processing	21	Image pre-processing details	√		
	22	Resampling method and its parameters	√		
	23	Discretization method and its parameters	√		
	24	Image types (e.g., original, filtered, transformed)	√		
Feature extraction	25	Feature extraction method	√		
	26	Feature classes	√		
	27	Number of features	√		
	28	Default configuration statement for remaining parameters	√		
Data preparation	29	Handling of missing data	√		
	30	Details of class imbalance			n/a
	31	Details of segmentation reliability analysis	√		
	32	Feature scaling details (e.g., normalization, standardization)	√		
	33	Dimension reduction details	√		
Modeling	34	Algorithm details	√		
	35	Training and tuning details	√		
	36	Handling of confounders			n/a
	37	Model selection strategy	√		
Evaluation	38	Testing technique (e.g., internal, external)	√		

Section	No	Item	Yes	No	n/a or n/e
	39	Performance metrics and rationale for choosing	√		
	40	Uncertainty evaluation and measures (e.g., confidence intervals)	√		
	41	Statistical performance comparison (e.g., DeLong's test)		√	
	42	Comparison with non-radiomic and combined methods		√	
	43	Interpretability and explainability methods		√	
Results					
	44	Baseline demographic and clinical characteristics	√		
	45	Flowchart for eligibility criteria		√	
	46	Feature statistics (e.g., reproducibility, feature selection)	√		
	47	Model performance evaluation	√		
	48	Comparison with non-radiomic and combined approaches		√	
Discussion					
	49	Overview of important findings	√		
	50	Previous works with differences from the current study	√		
	51	Practical implications	√		
	52	Strengths and limitations (e.g., bias and generalizability issues)	√		
Open Science*					
Data availability	53	Sharing images along with segmentation data	√		
	54	Sharing radiomic feature data	√		

Section	No	Item	Yes	No	n/a or n/e
Code availability	55	Sharing pre-processing scripts or settings	√		
	56	Sharing source code for modeling	√		
Model availability	57	Sharing final model files	√		
	58	Sharing a ready-to-use system			n/e

Yes, details provided; **No**, details not provided; **n/e,** not essential; **n/a,** not applicable

*Open Science: All image data are publicly available (links provided in the manuscript); feature data with segmentation, script pipeline with models are available from the corresponding author.

Table S2: List of LIFEx (v7.2.3) features used for analysis

Feature class/matrix (no. of features)	Features within the class
**Shape (14)**	Volume, approximate volume (AppVolume) , voxels counting, surface area (SA), surface to volume ratio (SA/V), compacity, compactness1, compactness2, spherical disproportion, sphericity, asphericity, , centre of mass shift, maximum 3D diameter (Max3DDiameter), integrated intensity
**First order (54)**	**<u>Intensity based</u>**: Mean (I_Mean), variance (I_Variance), skewness (I_Skewness) , kurtosis (I_Kurtosis), median (I_Median), minimum gray level (I_MinGL), 10thpercentile (I_10thPCTL), 25th percentile (I_25thPCTL), 50thpercentile (I_50thPCTL), 75thpercentile (I_75thPCTL), 90thpercentile (I_90thPCTL), standard deviation (I_StDev), maximum gray level (I_MaxGL), interquartile range (I_IQR), range (I_Range), mean absolute deviation (I_MAD), robust mean absolute deviation (I_RobustMAD), median absolute deviation (I_MedianAD), coefficient of variation (I_COV), quartile coefficient of dispersion (I_QCOD), area under curveCsh (I_AUCCsh) , energy (I_Energy), root mean square (I_RMS), total lesion glycolysis (I_TLG)<br>**<u>Intensity-histogram based features</u>**: Mean (IH_Mean), Variance (IH_Variance), skewness (IH_Skewness), kurtosis (IH_Kurtosis), median (IH_Median), minimum gray level (IH_MinGL), 10th percentile (IH_10thPCTL), 25th percentile (IH_25thPCTL), 50th percentile (IH_50thPCTL), 75th percentile (IH_75thPCTL), 90th percentile (IH_90thPCTL), standard deviation (IH_StDev), maximum gray level (IH_MaxGL), mode (IH_Mode), interquarter range (IH_IQR), range (IH_Range), mean absolute deviation (IH_MAD), robust mean absolute deviation (IH_RobustMAD), median absolute deviation (IH_MedianAD), coefficient of variation (IH_COV), quartile coefficient of dispersion (IH_QuartileCOD), EntropyLog10 (IH_EntropyLog10), EntropyLog2

Feature class/matrix (no. of features)	Features within the class
	(IH_EntropyLog2), AreaUnderCurveCsh (IH_AUCCsh), uniformity (IH_Uniformity), root mean square (IH_RMS), maximum histogram gradient (IH_MaxHGr), maximum histogram gradient gray level (IH_MaxHGrGL), minimum histogram gradient (IH_MinHGr), minimum histogram gradient gray level(IH_MinHGrGL)
**Gray level cooccurrence matrix (GLCM) (24)**	Joint maximum (GLCM_JointMaximum), joint average (GLCM_JointAverage), joint variance (GLCM_JointVariance), joint entropylog2 (GLCM_JointEntropyLog2), joint entropy log10 (GLCM_JointEntropyLog10), difference average (GLCM_DifferenceAverage), difference variance (GLCM_DifferenceVariance), difference entropy (GLCM_DifferenceEntropy), sum average (GLCM_SumAverage), sum variance (GLCM_SumVariance), sum entropy (GLCM_SumEntropy), angular second moment (GLCM_AngularSecondMoment), contrast (GLCM_Contrast), dissimilarity (GLCM_Dissimilarity), inverse difference (GLCM_ID), normalised inverse difference (GLCM_NormalisedID), inverse difference moment (GLCM_IDMoment), normalised inverse difference moment (GLCM_NormalisedIDMoment), inverse variance (GLCM_InverseVariance), correlation (GLCM_Correlation), autocorrelation (GLCM_Autocorrelation), cluster tendency (GLCM_ClusterTendency), cluster shade (GLCM_ClusterShade), cluster prominence (GLCM_ClusterProminence)
**Gray level run length matrix (GLRLM) (11)**	Short run emphasis (GLRLM_SRE), long run emphasis (GLRLM_LRE), low gray level run emphasis (GLRLM_LGLRE), high gray level run emphasis (GLRLM_HGLRE), short run low gray level emphasis (GLRLM_SRLGLE), short run high gray level emphasis (GLRLM_SRHGLE), long run low gray level emphasis

Feature class/matrix (no. of features)	Features within the class
	(GLRLM_LRLGLE), long run high gray level emphasis (GLRLM_LRHGLE), gray level non uniformity (GLRLM_GLNU), run length non uniformity (GLRLM_RLNU), run percentage (GLRLM_RP)
**Gray level size zone matrix (GLSZM) (16)**	Small zone emphasis (GLSZM_SZE), large zone emphasis (GLSZM_LZE), low gray level zone emphasis (GLSZM_LGLZE), high gray level zone emphasis (GLSZM_HGLZE), small zone low gray level emphasis (GLSZM_SZLGLE), small zone high gray level emphasis (GLSZM_SZHGLE), large zone low gray level emphasis (GLSZM_LZLGLE), large zone high gray level emphasis (GLSZM_LZHGLE), gray level non uniformity (GLSZM_GLNU), normalised gray level non uniformity (GLSZM_NGLNU), zone size non uniformity (GLSZM_ZSNU), normalised zone size non uniformity (GLSZM_NormalisedZSNU), zone percentage (GLSZM_ZP), gray level variance (GLSZM_GLV), zone size variance (GLSZM_ZSV), zone size entropy (GLSZM_ZSEntropy)
**Neighborhood gray-tone difference matrix (NGTDM) (5)**	Busyness (NGTDM_Busyness), coarseness (NGTDM_Coarseness), complexity (NGTDM_Complexity), contrast (NGTDM_Contrast), strength (NGTDM_Strength)

Table S3: Summary statistics of radiomic features in dataset 1 (training set)

SN	Feature Class	Feature	Mean	Standard deviation (SD)	Median	Inter quartile range (IQR)
1	Shape	Volume	18711	20713	12894	13703
2	Shape	AppVolume	22201	19432	16288	19488
3	Shape	VoxelsCounting	347	304	254	304
4	Shape	SA	4116	2831	3336	2599
5	Shape	SA_V	0.273	0.0695	0.272	0.09
6	Shape	Compacity	3.91	1.08	3.67	1.28
7	Shape	Compactness1	0.0364	0.00435	0.037	0.0059
8	Shape	Compactness2	0.478	0.108	0.485	0.154
9	Shape	SphericalDisproportion	1.3	0.117	1.27	0.13
10	Shape	Sphericity	0.777	0.0632	0.786	0.085
11	Shape	Asphericity	0.296	0.117	0.272	0.138
12	Shape	CentreOfMassShift	0.924	0.658	0.774	0.741
13	Shape	Max3DDiameter	47.1	14.2	44.3	16.2
14	Shape	IntegratedIntensity	95691	180514	44901	83951
15	First-order	I_Mean	4.19	2.18	3.71	3.12
16	First-order	I_Variance	4.44	5.83	2.32	5.282
17	First-order	I_Skewness	0.515	0.466	0.475	0.487
18	First-order	I_Kurtosis	-0.279	1.03	-0.517	0.7271
19	First-order	I_Median	3.88	2.06	3.47	2.81
20	First-order	I_MinGL	1.19	0.765	1.03	1.031
21	First-order	I_10thPCTL	2.1	1.17	1.9	1.57
22	First-order	I_25thPCTL	2.76	1.46	2.51	1.88
23	First-order	I_50thPCTL	3.88	2.06	3.47	2.81
24	First-order	I_75thPCTL	5.42	3	4.71	4.13
25	First-order	I_90thPCTL	6.81	3.71	6.06	5.53
26	First-order	I_StDev	1.8	1.11	1.52	1.526
27	First-order	I_MaxGL	8.9	4.83	8.26	7.32

SN	Feature Class	Feature	Mean	Standard deviation (SD)	Median	Inter quartile range (IQR)
28	First-order	I_IQR	2.67	1.86	2.21	2.27
29	First-order	I_Range	7.72	4.52	6.8	6.61
30	First-order	I_MAD	1.49	0.944	1.26	1.262
31	First-order	I_RobustMAD	1.11	0.745	0.933	0.936
32	First-order	I_MedianAD	1.46	0.925	1.25	1.207
33	First-order	I_COV	0.426	0.126	0.418	0.153
34	First-order	I_QCOD	0.319	0.105	0.304	0.145
35	First-order	I_AUCCsh	1.47	0.681	1.33	0.971
36	First-order	I_Energy	12620	30259	3816	9268
37	First-order	I_RMS	4.58	2.4	3.99	3.44
38	First-order	I_TLG	107	151	58.3	87.2
39	First-order	IH_Mean	13.9	6.96	12.4	10.02
40	First-order	IH_Variance	44.9	54.3	23.7	54.51
41	First-order	IH_Skewness	0.509	0.461	0.457	0.491
42	First-order	IH_Kurtosis	-0.287	1.01	-0.526	0.7645
43	First-order	IH_Median	13	6.57	12	9
44	First-order	IH_MinGL	4.29	2.46	4	4
45	First-order	IH_10thPCTL	7.22	3.74	7	5.25
46	First-order	IH_25thPCTL	9.31	4.67	8.5	6
47	First-order	IH_50thPCTL	13	6.57	12	9
48	First-order	IH_75thPCTL	17.8	9.58	15.5	13
49	First-order	IH_90thPCTL	22.3	11.7	20	17.2
50	First-order	IH_StDev	5.74	3.46	4.87	4.91
51	First-order	IH_MaxGL	28.8	14.9	27	24
52	First-order	IH_Mode	10.8	7.54	9	7
53	First-order	IH_IQR	8.53	5.94	7	7.2
54	First-order	IH_Range	24.5	13.9	21.5	22
55	First-order	IH_MAD	4.76	2.96	4.06	4.05
56	First-order	IH_RobustMAD	3.71	2.42	3.14	3.02

SN	Feature Class	Feature	Mean	Standard deviation (SD)	Median	Inter quartile range (IQR)
57	First-order	IH_MedianAD	4.66	2.91	4	3.82
58	First-order	IH_COV	0.407	0.118	0.397	0.143
59	First-order	IH_QuartileCOD	0.305	0.105	0.297	0.142
60	First-order	IH_EntropyLog10	1.21	0.275	1.24	0.36
61	First-order	IH_EntropyLog2	4.03	0.914	4.11	1.22
62	First-order	IH_AUCCsh	4.34	2.17	3.86	3.12
63	First-order	IH_Uniformity	0.0889	0.0785	0.067	0.0583
64	First-order	IH_RMS	0.0195	0.0119	0.0165	0.0139
65	First-order	IH_MaxHGr	14.1	22.3	8.5	10.6
66	First-order	IH_MaxHGrGL	7.44	6.49	6	6
67	First-order	IH_MinHGr	-10.7	13.9	-7	7
68	First-order	IH_MinHGrGL	14.5	8.48	13	9
69	GLCM	GLCM_JointMaximum	0.0332	0.0612	0.0158	0.02262
70	GLCM	GLCM_JointAverage	15.3	7.69	13.6	10.95
71	GLCM	GLCM_JointVariance	45.6	53.7	25.5	55.1
72	GLCM	GLCM_JointEntropyLog2	7.81	1.73	7.97	2.26
73	GLCM	GLCM_JointEntropyLog10	2.35	0.52	2.4	0.68
74	GLCM	GLCM_DifferenceAverage	4.37	2.34	4.2	3.28
75	GLCM	GLCM_DifferenceVariance	15.2	14.9	9.88	18.27
76	GLCM	GLCM_DifferenceEntropy	7.81	1.73	7.97	2.26
77	GLCM	GLCM_SumAverage	30.5	15.4	27.1	21.9
78	GLCM	GLCM_SumVariance	127	161	64	143.5
79	GLCM	GLCM_SumEntropy	7.28	2.13	7.64	2.52
80	GLCM	GLCM_AngularSecondMoment	0.0151	0.0376	0.00538	0.00957
81	GLCM	GLCM_Contrast	39.8	38.5	27.5	46.3
82	GLCM	GLCM_Dissimilarity	4.37	2.34	4.2	3.28
83	GLCM	GLCM_ID	0.346	0.126	0.312	0.147
84	GLCM	GLCM_NormalisedID	0.864	0.0211	0.862	0.029
85	GLCM	GLCM_IDMoment	0.264	0.142	0.221	0.152

SN	Feature Class	Feature	Mean	Standard deviation (SD)	Median	Inter quartile range (IQR)
86	GLCM	GLCM_NormalisedIDMoment	0.956	0.0126	0.956	0.017
87	GLCM	GLCM_InverseVariance	0.0123	0.0139	0.00714	0.01241
88	GLCM	GLCM_Correlation	0.489	0.133	0.503	0.174
89	GLCM	GLCM_Autocorrelation	318	309	193	362.6
90	GLCM	GLCM_ClusterTendency	127	161	64	143.5
91	GLCM	GLCM_ClusterShade	733	2673	58.4	655.98
92	GLCM	GLCM_ClusterProminence	119658	338976	13345	89325
93	GLRM	GLRLM_SRE	0.94	0.052	0.955	0.037
94	GLRM	GLRLM_LRE	1.32	0.434	1.21	0.2
95	GLRM	GLRLM_LGLRE	0.0334	0.0925	0.0112	0.01751
96	GLRM	GLRLM_HGLRE	284	268	177	329.2
97	GLRM	GLRLM_SRLGLE	0.0281	0.0691	0.0108	0.01673
98	GLRM	GLRLM_SRHGLE	272	258	170	321.3
99	GLRM	GLRLM_LRLGLE	0.0715	0.281	0.0136	0.02361
100	GLRM	GLRLM_LRHGLE	350	440	223	366
101	GLRM	GLRLM_GLNU	21.4	18	15.4	15.8
102	GLRM	GLRLM_RLNU	278	249	204	247
103	GLRM	GLRLM_RP	0.922	0.0617	0.939	0.049
104	NGTDM	NGTDM_Coarseness	0.0314	0.0185	0.0255	0.0248
105	NGTDM	NGTDM_Contrast	0.304	0.204	0.263	0.258
106	NGTDM	NGTDM_Busyness	0.699	2.69	0.188	0.263
107	NGTDM	NGTDM_Complexity	1224	1671	500	1547
108	NGTDM	NGTDM_Strength	7.7	6.78	5.86	8.67
109	GLSZM	GLSZM_SZE	0.579	0.154	0.605	0.185
110	GLSZM	GLSZM_LZE	289	1581	12.2	31.78
111	GLSZM	GLSZM_LGLZE	0.0383	0.102	0.0116	0.02034
112	GLSZM	GLSZM_HGLZE	275	248	189	330.3
113	GLSZM	GLSZM_SZLGLE	0.0157	0.039	0.00669	0.00875
114	GLSZM	GLSZM_SZHGLE	185	189	115	238.8

SN	Feature Class	Feature	Mean	Standard deviation (SD)	Median	Inter quartile range (IQR)
115	GLSZM	GLSZM_LZLGLE	107	793	0.128	0.7594
116	GLSZM	GLSZM_LZHGLE	8052	37457	2577	3332
117	GLSZM	GLSZM_GLNU	6.86	4.16	5.78	4.71
118	GLSZM	GLSZM_NGLNU	0.0823	0.0697	0.0623	0.0571
119	GLSZM	GLSZM_ZSNU	59.6	76	31.9	61.1
120	GLSZM	GLSZM_NormalisedZSNU	0.354	0.123	0.352	0.175
121	GLSZM	GLSZM_ZP	0.41	0.189	0.437	0.294
122	GLSZM	GLSZM_GLV	43.4	45.5	26.8	51.4
123	GLSZM	GLSZM_ZSV	145	752	6.7	22.16
124	GLSZM	GLSZM_ZSEntropy	5.26	0.96	5.4	1.07

Table S4: Hyperparameters for the best model used for external validation for all outcomes

Outcome	Model	Hyperparameters
All-cause mortality	Penalized Cox	'random_state': 42, 'coxnetsurvivalanalysis__tol': 0.0001, 'coxnetsurvivalanalysis__n_alphas': 200, 'coxnetsurvivalanalysis__max_iter': 3, 'coxnetsurvivalanalysis__l1_ratio': 0.033, 'coxnetsurvivalanalysis__alphas': [0.0017]}
Loco-regional recurrence/residual disease	Random survival forest	'random_state': 40, 'oob_score': False, 'n_jobs': 2, 'n_estimators': 100, 'min_weight_fraction_leaf': 0.19, 'min_samples_split': 4, 'min_samples_leaf': 2, 'max_leaf_nodes': 16, 'max_features': 'sqrt', 'max_depth': 2, 'bootstrap': False
Distant metastasis	Penalized Cox	'random_state': 42, ‘coxnetsurvivalanalysis__tol': 0.0001, 'coxnetsurvivalanalysis__n_alphas': 500, 'coxnetsurvivalanalysis__max_iter': 3, 'coxnetsurvivalanalysis__l1_ratio': 0.033, 'coxnetsurvivalanalysis__alphas': [0.0398]

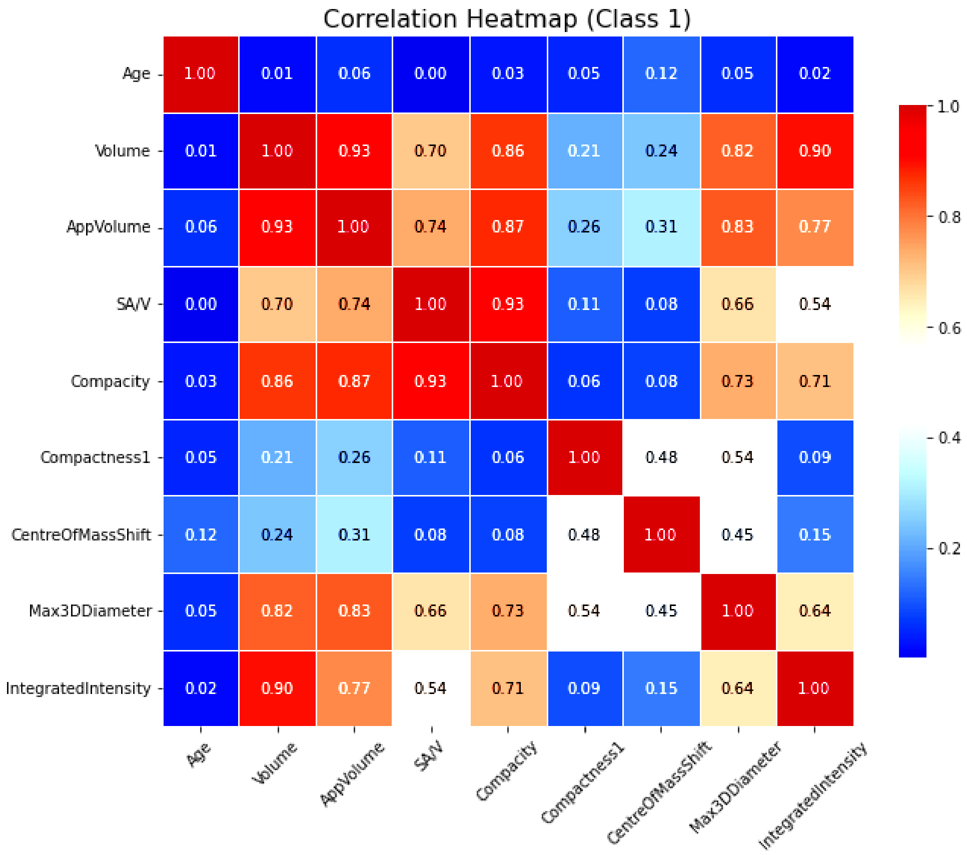

Figure S1: Correlation heatmap for age and shape-based features

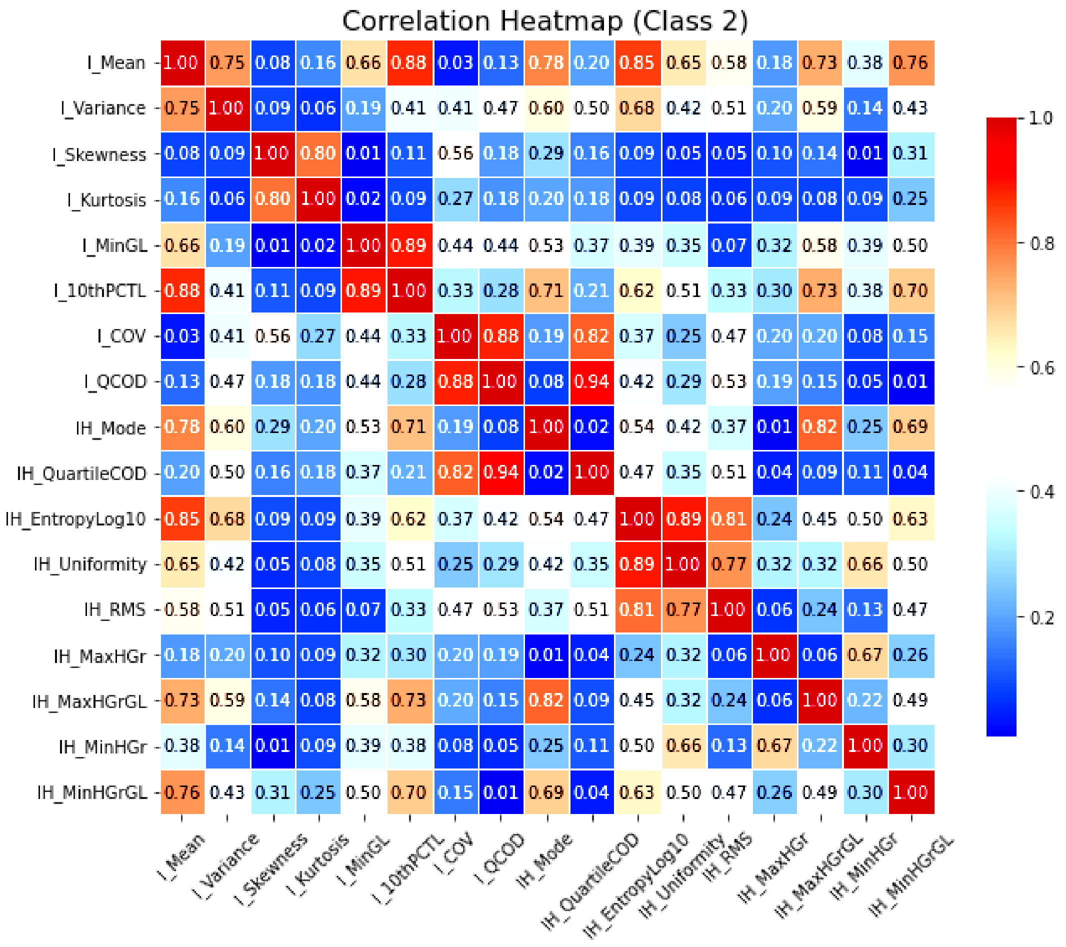

Figure S2: Correlation heatmap for first-order features

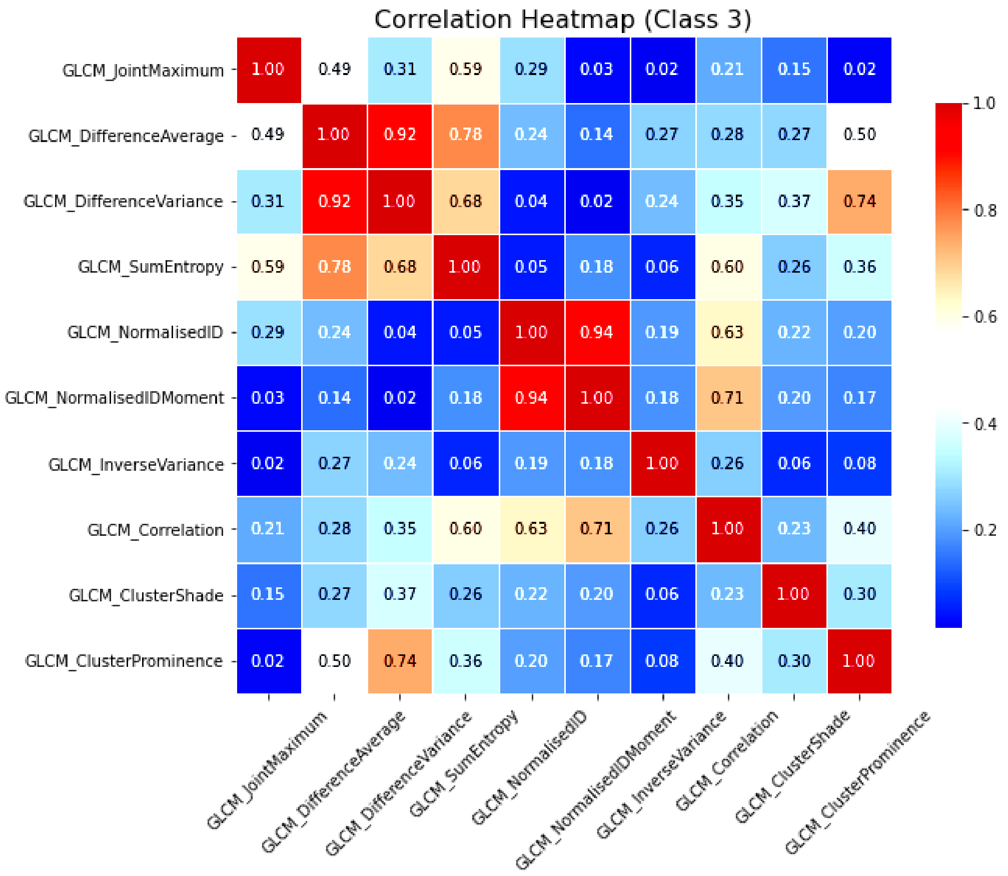

Figure S3: Correlation heatmap for gray level co-occurrence matrix (GLCM) features

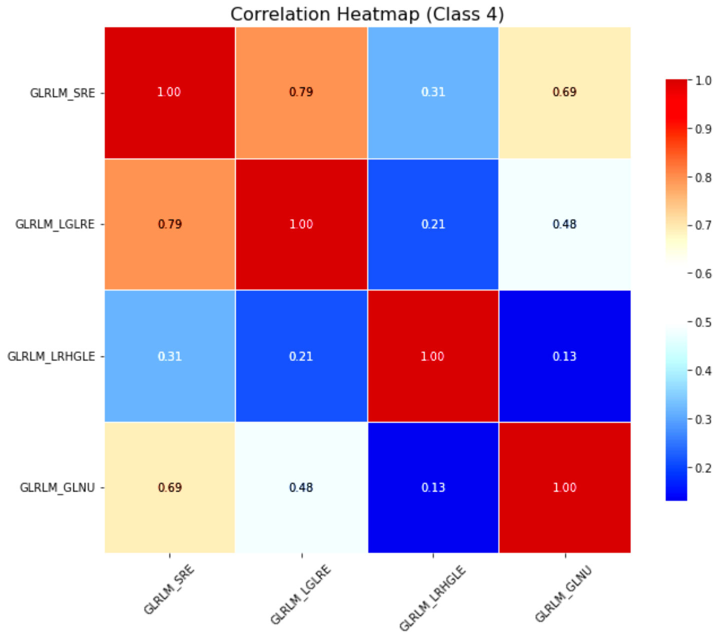

Figure S4: Correlation heatmap for gray level run length matrix (GLRLM) features

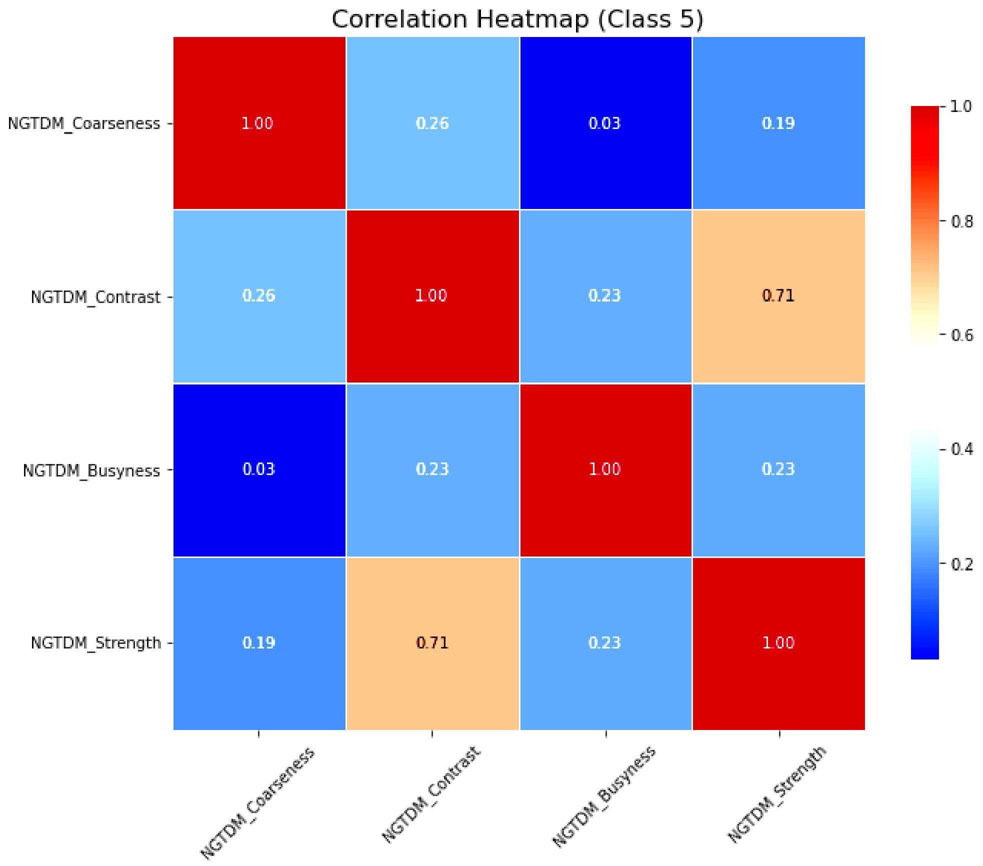

Figure S5: Correlation heatmap for neighborhood gray-tone difference matrix (NGTDM) features

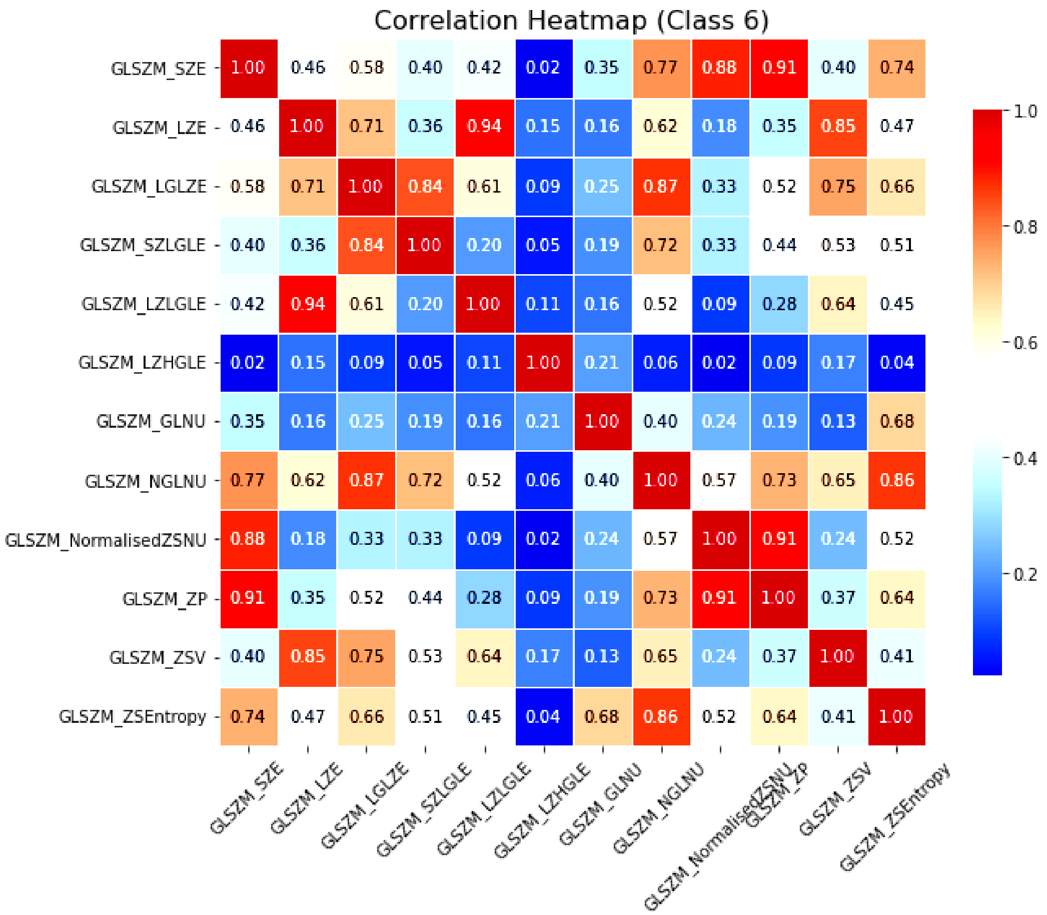

Figure S6: Correlation heatmap for gray level size zone matrix (GLSZM) features
